# Biomass Valorization through Catalytic Pyrolysis Using Metal-Impregnated Natural Zeolites: From Waste to Resources

**DOI:** 10.3390/polym16131912

**Published:** 2024-07-04

**Authors:** Diego Venegas-Vásconez, Lourdes Orejuela-Escobar, Alfredo Valarezo-Garcés, Víctor H. Guerrero, Luis Tipanluisa-Sarchi, Serguei Alejandro-Martín

**Affiliations:** 1 Departamento de Ingeniería de Maderas, Universidad del Bío-Bío, Concepción 4081112, Chile; diego.venegas1801@alumnos.ubiobio.cl; 2 Laboratorio de Cromatografía Gaseosa y Pirólisis Analítica, Universidad del Bío-Bío, Concepción 4081112, Chile; 3 Departamento de Ingeniería Química, Universidad San Francisco de Quito USFQ, Diego de Robles s/n y Av. Interoceánica, Quito 170157, Ecuador; lorejuela@usfq.edu.ec; 4 Departamento de Ingeniería Mecánica, Universidad San Francisco de Quito USFQ, Diego de Robles s/n y Av. Interoceánica, Quito 170157, Ecuador; avalarezo@usfq.edu.ec; 5 Departamento de Materiales, Escuela Politécnica Nacional, Quito 170525, Ecuador; victor.guerrero@epn.edu.ec; 6 Facultad de Mecánica, Escuela Superior Politécnica de Chimborazo, Riobamba 060155, Ecuador; luis.tipanluisa@espoch.edu.ec

**Keywords:** biomass valorization, pyrolysis, natural zeolite, transition metal, BTX

## Abstract

Catalytic biomass pyrolysis is one of the most promising routes for obtaining bio-sustainable products that replace petroleum derivatives. This study evaluates the production of aromatic compounds (benzene, toluene, and xylene (BTX)) from the catalytic pyrolysis of lignocellulosic biomass (*Pinus radiata* (PR) and *Eucalyptus globulus* (EG)). Chilean natural zeolite (NZ) was used as a catalyst for pyrolysis reactions, which was modified by double ion exchange (H2NZ) and transition metals impregnation (Cu5H2NZ and Ni5H2NZ). The catalysts were characterized by nitrogen adsorption, X-ray diffraction (XRD), ammonium programmed desorption (TPD-NH_3_), and scanning electron microscopy with energy dispersive X-ray spectroscopy (SEM-EDS). Analytical pyrolysis coupled with gas chromatography/mass spectrometry (Py-GC/MS) allowed us to study the influence of natural and modified zeolite catalysts on BTX production. XRD analysis confirmed the presence of metal oxides (CuO and NiO) in the zeolite framework, and SEM-EDS confirmed successful metal impregnation (6.20% for Cu5H2NZ and 6.97% for Ni5H2NZ). Py-GC/MS revealed a reduction in oxygenated compounds such as esters, ketones, and phenols, along with an increase in aromatic compounds in PR from 2.92% *w*/*w* (without catalyst) to 20.89% *w*/*w* with Ni5H2NZ at a biomass/catalyst ratio of 1/5, and in EG from 2.69% *w*/*w* (without catalyst) to 30.53% *w*/*w* with Ni5H2NZ at a biomass/catalyst ratio of 1/2.5. These increases can be attributed to acidic sites within the catalyst pores or on their surface, facilitating deoxygenation reactions such as dehydration, decarboxylation, decarbonylation, aldol condensation, and aromatization. Overall, this study demonstrated that the catalytic biomass pyrolysis process using Chilean natural zeolite modified with double ion exchange and impregnated with transition metals (Cu and Ni) could be highly advantageous for achieving significant conversion of oxygenated compounds into hydrocarbons and, consequently, improving the quality of the condensed pyrolysis vapors.

## 1. Introduction

Non-renewable fossil resources such as natural gas, oil, and coal have been the dominant chemical and energy supply for several decades. Global fossil fuel consumption increased from 40,000 terawatt-hours (TWh) in 1965 to 136,000 TWh in 2019 [1]. Chemical production also depends on fossil fuels. It is estimated that 12% of the world’s oil is currently dedicated to producing chemicals that are essential precursors of value-added products such as fertilizers, plastics, rubber, fibers, and solvents [2]. Therefore, sustainable alternatives are required to meet the world’s energy needs. One of the renewable sources that can fulfill this role is biomass due to its availability, compatibility with current energy infrastructure, and versatility in various applications.

Consequently, numerous studies have explored using agricultural and forestry residues, livestock manure, municipal waste, organic sewage, and industrial refuse as substitutes or oil in their present uses [3]. Among these alternatives, lignocellulosic biomass (LCB) is particularly noteworthy, with an annual production rate exceeding 180 billion tons, making it a promising and standout source [2]. In 2019, Chile had a forest area of 17 million hectares, of which 14.7 million hectares were native forests and 2.3 million were forest plantations. *Pinus radiata* and *Eucalyptus globulus*, with 92.66% of the total, were the most planted species, yielding between 25 and 35 cubic meters per hectare per year [4]. Additionally, silvicultural activities and the processing of forest resources generate more than 4 million metric tons of energetically exploitable residues [5].

Biomass can undergo various technical conversion processes to generate different forms of energy. Among these methods, thermochemical conversion techniques such as pyrolysis and hydrothermal liquefaction provide a practical means of producing liquid fuels. These liquid byproducts, called bio-oils, are considered viable alternatives to traditional petroleum fuels for power generation, heating, or extracting valuable chemicals [6]. Fast pyrolysis of biomass with a rapid heating rate (>500 °C s^−1^) to intermediate temperatures (400–600 °C) is a promising way to generate bio-oil from the fast decomposition of biomass in the absence of oxygen, by which the short vapor residence time can lead to a high bio-oil yield with few other products like gas and solid char [7]. However, the bio-oil produced from pyrolysis is generally an aqueous and highly oxygenated mixture of phenols, acids, and a fraction of hydrocarbons. Furthermore, its high acidity, low stability, corrosive nature, high viscosity, and low vapor pressure are other significant limitations that restrict its direct application in various uses. Therefore, bio-oil upgrading is essential to transform it into a product that can be used and marketed [8]. 

There are two primary catalytic routes to reduce the oxygen content in bio-oil. The first method, catalytic hydroprocessing, encompasses hydrocracking and hydrodeoxygenation [9]. This approach offers the advantage of producing high-quality bio-oil with low oxygen levels and a high H/C ratio. However, it requires a significant hydrogen supply, making the process economically costly [8]. The second approach involves catalytic cracking of oxygenated compounds. This process may offer a more cost-effective alternative and could be preferred for upgrading bio-oil [10]. The pyrolysis process can be executed in two configurations in catalytic cracking, depending on the catalyst’s introduction. The first configuration, termed in situ pyrolysis, entails blending a catalyst with the biomass. Conversely, the second configuration (ex situ pyrolysis) placed the catalyst downstream from the biomass, enabling the resulting pyrolytic vapors to traverse the catalyst bed. Consequently, the catalyst will only come into contact with the volatilized gases [11]. In in situ pyrolysis, the generated pyrolytic vapors cannot come into contact with a significant amount of catalysts, thus requiring a higher biomass/catalyst ratio to achieve better deoxygenation activity [12]. In the catalytic pyrolysis of hybrid poplar, Wang et al. [13] used an HZSM-5 zeolite as a catalyst, finding that aromatics formed from ex situ pyrolysis were predominantly monocyclic aromatics, whereas in situ pyrolysis favored polycyclic aromatics. 

Using zeolite-based catalysts decomposes and dehydrates biomass polymers due to their high selectivity towards producing aromatic compounds and their ability to limit the formation of unwanted products, such as carboxylic acids and oxygenated compounds [14]. In this context, renewable aromatic compounds are crucial foundational components capable of partially substituting petrochemical resources in producing biofuels and chemicals. They have the potential to diminish reliance on oil and mitigate environmental repercussions by emitting fewer SOx and CO_2_ emissions, thus fostering the growth of sustainable economies [15]. Synthetic zeolites (Y, ZSM-5, and H-ZSM-5) are the preferred catalysts in pyrolysis oil upgrading processes, but they have a limitation due to their high cost (e.g., ZSM-5 → 120 USD·kg^−1^, Y → 180 USD·kg^−1^). In contrast, natural zeolites are inexpensive and abundant; for example, natural clinoptilolite has an average value of 242 USD· metric ton^−1^ [16]. Besides the lower cost of extraction, natural zeolites have some advantages over synthetic zeolites, like reduced environmental impact in their extraction, as synthetic zeolite production involves chemicals and intensive industrial processes. Additionally, the production of natural zeolites generally requires less energy than zeolite synthesis, which can result in a lower energy footprint. Finally, natural zeolites require less post-processing for industrial applications, simplifying preparation procedures and reducing associated costs [17,18].

The drawback of natural zeolites is their lower performance in pyrolysis compared to synthetic ones, which can be significantly improved through appropriate treatments [19]. The ionic exchange procedure enhances Brönsted acid sites (bridging hydroxyls connected to aluminum atoms in the framework) within the internal structure of zeolites, leading to bio-oil deoxygenation [20]. Additionally, the impregnation of metals and metal oxides into zeolites reduces the formation of acids and oxygenated compounds in catalytic pyrolysis since the metal particles can provide sites for the formation and breaking of C-C bonds and the acid sites of the zeolites can act in the isomerization of olefins [21]. In a study conducted by Veses et al. [22], the performance of various metal cations/ZSM-5 catalysts in bio-oil upgrading through ex situ pyrolysis of biomass was compared. They demonstrated that Ni/ZSM-5 and Cu/ZSM-5 catalysts outperformed Mg/ZSM-5 and H-ZSM-5 catalysts. Expressly, the results indicated that Ni/ZSM-5 and Cu/ZSM-5 yielded bio-oil containing approximately 35% and 31% hydrocarbons, respectively, while Mg/ZSM-5 and H-ZSM-5 catalysts produced bio-oil samples with 29% hydrocarbons. Rajić et al. [23] compared the catalytic activity of clinoptilolite containing NiO, ZnO, and Cu_2_O nanoparticles in the pyrolysis of hardwood lignin. They found that the production of phenolic compounds primarily depended on the metal cations in the zeolite and not on the type of acid sites present. The bio-oil produced contained phenols in the range of 39% for clinoptilolite with ZnO, 50% for clinoptilolite with Cu_2_O, and 54% for clinoptilolite with NiO. They attribute the higher yield of phenols in hardwood pyrolysis to using clinoptilolite with NiO as a catalyst for the interaction between the metal nanoparticles and the zeolite during the reaction. Regardless of the prominent results obtained, the significant role of the catalyst in pyrolysis product distribution is inconclusive and requires further analysis to elucidate the reaction pathways induced by the catalyst addition (clinoptilolite).

Despite the extensive literature on the effect of synthetic zeolite-based catalysts in lignocellulosic biomass pyrolysis, there is currently no consensus on the influence of natural zeolite application as their replacement. Previous evidence indicates that the formation of BTX is promoted due to the synergy between lignocellulosic biomass components and zeolite-based catalysts. Therefore, this study aims to evaluate the composition of compounds formed during the catalytic pyrolysis of *Pinus radiata* and *Eucalyptus globulus* from the Bio-Bio Region, Chile, using Chilean natural zeolite impregnated with transition metals (Cu and Ni) and their influence on the formation of aromatic compounds: benzene, toluene, and xylene (BTX), as well as the reduction in oxygenated compounds.

## 2. Materials and Methods

### 2.1. Materials

#### 2.1.1. Biomass

Investigaciones Forestales Bioforest S.A., based in Bío Bío, Chile, supplied biomass samples (PR and EG) as wood chips. These chips were composed entirely of wood without bark, and the specific location of the sample within the tree trunk (sapwood/heartwood) was not considered. The samples were ground and sieved to attain an average particle size of 0.33 mm. Following a previously documented procedure, they were stored in a desiccator until further use [24].

#### 2.1.2. Catalyst

Chilean natural zeolite (53% clinoptilolite, 40% mordenite, and 7% quartz) was provided by “Minera Formas”. The zeolite sample was ground and sieved with an average particle size of 0.3 mm, and then rinsed with ultrapure water, oven-dried at 125 °C for 24 h, and finally stored in a desiccator until further use [25].

### 2.2. Methods

#### 2.2.1. Catalyst Modification

The Chilean natural zeolite (NZ) was modified by ion exchange using ammonium sulphate [(NH_4_)_2_ SO_4_] (0.1 mol∙dm^−3^), according to the previous work reported by Alejandro et al. [26]. The exchanged zeolite was denoted as (H2NZ). Finally, the sample was dried in an oven at 125 °C for 24 h and subjected to thermal degassing with a flow of N_2_ inert gas (100 cm^3^∙min^−1^) for two hours with a heating rate of 10 °C∙min^−1^. The H2NZ sample was impregnated with transition metals (Cu and Ni) using metal salts precursors: nickel nitrate Ni(NO_3_)_2_·6H_2_O and copper nitrate Cu(NO3)_2_·3H_2_O, through the incipient wet impregnation technique, as reported previously [27] and considering a metallic salts concentration of 0.05 mol∙dm^−3^ [28,29]. Finally, the samples were calcined at 500 °C for 4 h and then cooled until reaching room temperature. Such samples were named Cu5H2NZ and Ni5H2NZ, respectively.

#### 2.2.2. Biomass Characterization

The elemental analysis of PR and EG samples followed the ASTM D5373 Standard method [30] and was conducted using a Leco CHNS 628 elemental analyzer (St. Joseph, MI, USA). Proximate analyses, which included determining moisture content, volatile matter, ash content, and fixed carbon, were carried out in a muffle furnace, adhering to the ASTM D3172 Standard method [31]. The oxygen (O) content was calculated by the difference between carbon (C), hydrogen (H), and nitrogen (N) measurements.

Around 300–400 mg of PR and EG with 45–60 mesh were considered to quantify extractives, using a Soxhlet system with an acetone/water solution (9/1) for 16 h, following the TAPPI T280 pm-99 method [32], under the procedure described by Aguayo et al. [33]. The methods for determining holocellulose, α-cellulose, and lignin can be found in previous research [34].

#### 2.2.3. Physico-Chemical Characterization of Natural and Modified Zeolite Samples

Both natural and modified zeolites were characterized using various physico-chemical techniques. X-ray powder diffraction (XRD) analysis was used to confirm the modifications of the zeolite structure. X-ray diffraction patterns were obtained using a Bruker D8 Advance equipment (Billerica, MA, USA) operated at 20 mA and 40 kV with a copper cathode lamp (*λ* = 1.541 Å). XRD patterns were collected in the angular range 6–70°, a step size of 0.02 and a time/step of 0.2 s. The Scherrer equation (Equation (1)) [35] can be estimated from the width of the X-ray diffraction peaks

(1) $$D=\frac{0.9\lambda }{B\;cos\theta }$$

where *D* = Average crystal size, *λ* = X-ray wavelength (0.154 nm), *θ* = Bragg angle, and *B* = FWHM in radians.

In order to compare the crystallinity from the natural and modified zeolites, the relative crystallinity was determined according to ASTM D5357 [36] recommendations (Equation (2)). This value compares the peak intensity corresponding to the planes of the crystalline zeolite with the modified zeolite peaks [37].

(2) $$Crystallinity=\frac{S_{sample}}{S_{reference}}\times 100$$

where *crystallinity* = relative crystallinity (%), *S_sample_* = sum of integral peak intensities for the sample, and *S_reference_* = sum of integral peak intensities for the reference.

The surface morphology of the natural and modified zeolites was examined by SEM-JEOL JSM-IT300 scanning electron microscopy (JEOL, Tokyo, Japan) at 5–20 kV and 50 Pa. Energy-dispersed X-ray spectroscopy (EDS) was performed to confirm the presence of the impregnated transition metals on the surface.

Specific surface areas were determined by nitrogen adsorption and desorption at 77 K using a Micromeritics Gemini 2370 apparatus (Norcross, GA, USA), using 0.57 g of samples previously outgassed under nitrogen flow at 623 K for 2 h. The total area was calculated using the Brunauer–Emmett–Teller (BET) model with the Quantachrome, NOVATOUCH LX1 model. The total pore volume was obtained at P/P_0_ = 0.99. Micropore area and micropore volume were calculated by the t-plot method using the adsorption data range of 0.2 < P/P_0_ < 0.6. The pore size distribution was derived from the adsorption branch data of each isotherm using the Barrett–Joyner–Halenda (BJH) method [38].

The TGA experiments were carried out in a TGA Q500 thermogravimetric analyzer (TA Instruments, New Castle, DE, USA) to evaluate the thermal stability of the natural and modified zeolite. Approximately 10 ± 1 mg of the sample was placed on the thermobalance and heated from room temperature to 650 °C at a heating rate of 10 °C∙min^−1^. The experiments were conducted at atmospheric pressure with an N2 flow (50 mL∙min^−1^).

Ammonia thermal programmed desorption (NH_3_-TPD) contributes to the investigation of acid sites of the zeolite samples [39]. Samples (0.055 g) were degassed under nitrogen flow (0.05 L∙min^−1^), heated to 550 °C at 10 °C∙min^−1^, and finally cooled down to 125 °C to saturated with ammonia. After the samples were heated up to 550 °C at 10 °C∙min^−1^ in N2 (0.1 L∙min^−1^), the TPD system used a thermal conductivity detector (TCD) to determine the desorbed ammonia. The changes in conductivity vs. temperature were recorded since the temperature ranges in which NH_3_ desorbs are directly related to the strength of the acid sites of the zeolite, and the amount of chemisorbed NH_3_ is proportional to the number of acid sites per unit mass of the adsorbent [40]. 

#### 2.2.4. Catalytic Fast Pyrolysis of Biomass

The catalytic biomass pyrolysis was carried out in an analytical micro-pyrolysis reactor (Pyroprobe 5200HPR, CDS Analytical Co., Ltd., Oxford, PA, USA) connected in line with a gas chromatograph equipped with a mass spectrometer system, GC/MS (Clarus 690, QS8. Perkin Elmer, Waltham, MA, USA). The pyrolysis reactor consisted of a quartz tube heated by a platinum filament. Approximately 0.5 ± 0.1 mg of each sample was weighed by a microbalance (AD 6000 Ultra MicroBalance Perkin Elmer, Waltham, MA, USA). According to previous analyses, the experiments were conducted at 550 °C to guarantee maximum biomass decomposition and to achieve the highest pyrolysis oil yield [41]. He (pure 99.996%, BOC) was used as a carrier gas. The compounds in the pyrolysis-evolved gas were identified by referencing the NIST 2017 library and TurboMass 6.1.0 software. The ex situ configuration was chosen in the catalytic pyrolysis experiments [42]. In this configuration, the catalyst has no contact with the biomass, which allows for a more significant interaction between the pyrolysis vapors and the active sites of the catalysts. Additionally, Table 1 shows the conditions of catalytic biomass pyrolysis.

A semi-quantitative approach was employed to estimate the concentration of aromatic compounds, utilizing the ratio of absolute peak area to sample mass (as depicted in Equation (3)) [43].

(3) $$Yield\;of\;specific\;BTX\;compound=\frac{Peak\;area\;(a.u.)}{Sample\;mass\;(mg)}$$

The selective yield of individual BTX compounds (benzene, toluene, and xylene) within the total aromatic compounds was determined using Equation (4) [44].

(4) $$S_{AC}=\frac{Yield\;of\;specific\;BTX\;compound}{Total\;yield\;of\;aromatic\;compounds}$$

## 3. Results

### 3.1. Biomass Characterization

Table 2 presents the raw materials’ proximate analysis, ultimate analysis, and chemical composition of biomass samples (PR and EG). The proximate analysis revealed no significant differences among these wood species, indicating a high volatile content that supports their suitability for pyrolysis. Notably, the ash content in these samples was below 1%, eliminating concerns about the potential catalytic effects of ashes during pyrolysis [45]. 

The ultimate analysis poses the PR and EG at the same level as other woody biomass previously reported in the literature [46]. The high carbon (48.02–47.76%) and low nitrogen (0.29–0.09%) contents further affirm the appropriateness of PR and EG for thermochemical conversion processes and suggest that this raw material is unlikely to generate NOx and SOx emissions.

The obtained chemical composition results align with those reported by Wang et al. [47]. In softwoods (e.g., pine), cellulose content ranges from 40–44%, hemicellulose from 25–29%, lignin from 25–31%, and extractives from 1–5%. In hardwoods (e.g., eucalyptus), cellulose content ranges from 43–47%, hemicellulose from 25–35%, lignin from 16–24%, and extractives from 2–8%.

### 3.2. Physical-Chemical Characterization of Natural and Modified Zeolite Samples

#### 3.2.1. Crystallinity

**Figure 1:**
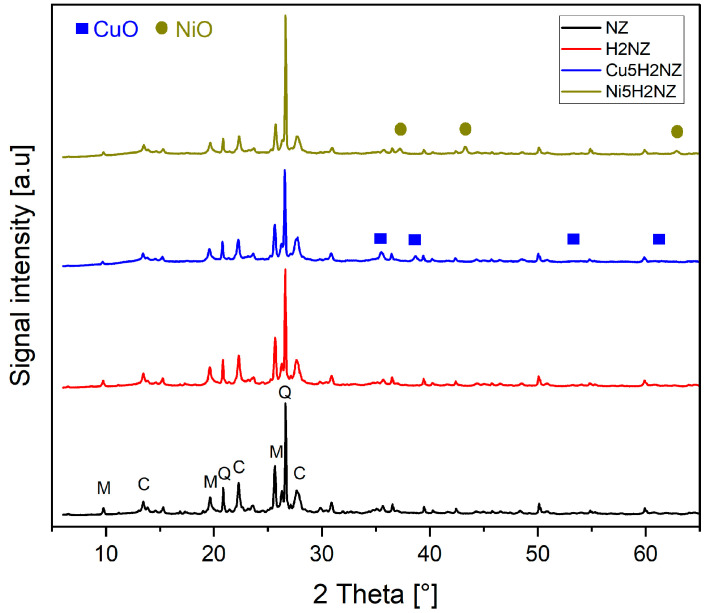
X-ray diffraction patterns for NZ, H2NZ, Cu5H2NZ, and Ni5H2NZ.

Figure 1 shows the X-ray diffraction patterns for metal (Cu, Ni) impregnated zeolites. It can be revealed in the XRD that the natural zeolite sample is highly crystalline, showing characteristic peaks of mordenite (M), clinoptilolite (C), and quartz (Q), which have been previously reported [23]. The characteristic peaks of quartz are observed at (21.0° and 26.7°), just as reported by Gurevich et al. [20]. It can be seen that the modifications made (double ion exchange and impregnation of transition metals) did not affect the crystallinity of the catalysts, as already reported by Trisunaryanti et al. [48]. 

The appearance of new peaks in the Cu5H2NZ and Ni5H2NZ diffractograms indicates the presence of the oxide metals on the zeolite surface and confirms the results obtained by SEM-EDS. Figure 1 shows a CuO phase at peaks of 35.10°, 39.30, 53.44°, and 61.40, which can be indexed to (002), (200), (020), and (−311) planes of crystalline CuO [8]. The peaks identified for nickel oxide (NiO) were found at positions 2θ: 37.25, 43.29, and 62.85 [49], which were indexed to (111), (200), and (220) planes of crystalline NiO. These results were consistent with the standard values of CuO and NiO and well-matched with International Centre for Diffraction Data (ICDD) reference codes 00-045-0937 and 01-089-7390. The metal oxides in the metal-modified zeolites were mainly dispersed on the surface of H2NZ and partially anchored in the pores of the catalysts, thus preserving the structure [50]. Thus, the low content of Cu and Ni doping reduced this agglomeration on the zeolite surface [51].

Using the Scherrer equation, crystal size can be estimated from the width of X-ray diffraction peaks. The results of crystal size measurements for each impregnated metal oxide phase are described in Table 3. The increase in crystal size is reflected in the decrease in pore size and surface area of the catalyst due to transition metal impregnation [35], and this is possible since the more impregnated metal into the pores of the carrier, the more the pores of the carrier with smaller fingers will be blocked [35].

Table 3 also shows the relative crystallinity of zeolites. The relative crystallinity of the natural and modified zeolite samples was calculated, confirming that the highest intensity was obtained in H2NZ, which was taken as the reference. During ion exchange, the cations present in the channels and cavities of the zeolite are replaced. This process can rearrange the crystalline structure, eliminating defects and improving the organization of the crystal lattice. This rearrangement can result in a more ordered structure and, consequently, an increase in the crystallinity. This might explain why the crystallinity of the ion-exchanged modified zeolite is higher than that of the natural zeolite [52]. The ion exchange process can also help eliminate impurities in the natural zeolite. These impurities may have caused distortions or defects in the original crystalline structure. By removing these impurities, the structure can become more homogeneous and ordered, which is reflected in a higher crystallinity index. Finally, ion exchange can improve the mobility of ions within the zeolite structure. Better ionic mobility can facilitate a more efficient rearrangement of atoms in the crystal lattice, promoting higher crystallinity [53,54,55]. The results demonstrate that the transition metals impregnation in zeolites (Cu5H2NZ and Ni5H2NZ) slightly decreased crystallinity compared to parent zeolites. However, this reduction did not damage the crystalline structure of the zeolite [50].

#### 3.2.2. Chemical Composition and Surface Morphology

Energy-dispersive X-ray spectroscopy (EDS) was conducted in order to identify the compensating cations (Na, K, Mg, Ca, Mg, Fe, and Ti) in the zeolite framework, as well as the impregnation of metals (Cu and Ni), and the results are shown in Table 4. The catalyst underwent an ion exchange process with NH_4_^+^, decreasing Na^+^, K^+^, and Ca^2+^ concentrations. Similar results were obtained in a previous work reported by Alejandro et al. [26]. These cations were found to have a lower affinity for the zeolite framework than NH_4_^+^. However, the Mg^2+^ cation was hardly affected by the ion exchange and had a higher affinity for clinoptilolite than NH_4_^+^ [56]. Generally, the surface concentration of compensation cations decreased due to replacing most of the alkaline and alkaline earth cations with NH_4_^+^, which later decomposed to H^+^ to generate Brönsted acid sites. Na ions in natural clinoptilolite zeolites are weakly bonded to the zeolite framework and are easy to remove, as confirmed by Ates and Hardacre [55]. During chemical modification, calcium cations move into the solution. In contrast, magnesium and potassium cations are less exchanged, probably due to the difficulty in accessing magnesium and potassium cations because of their larger radius compared to Ca^2+^ cations (for instance, Mg^2+^ has an atomic radius of 0.16 nm, while K^+^ and Ca^2+^ have atomic radii of 0.133 nm and 0.104 nm, respectively) [57]. The results of the copper and nickel-loaded samples show that metal impregnation indeed occurred and replaced only the ions of the compensation cations in the clinoptilolite phase. Similar results were observed by Rajić et al. [23].

**Figure 2:**
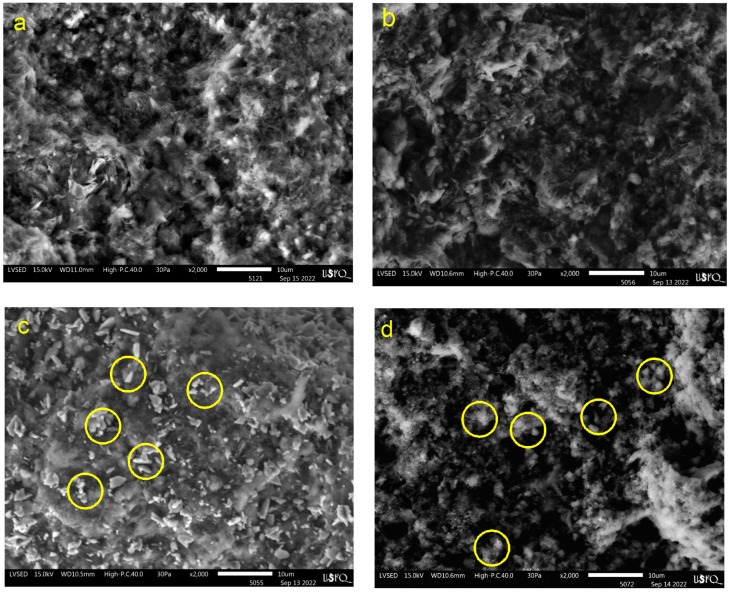
SEM images: (**a**) NZ, (**b**) H2NZ, (**c**) Cu5H2NZ, and (**d**) Ni5H2NZ.

Figure 2 shows the scanning electron microscope (SEM) images before and after impregnation with 2000X magnification. The natural zeolite (NZ) (Figure 2a) and natural with double ion exchange (H2NZ) (Figure 2b) present an irregular surface morphology without presenting significant changes between the zeolites. Figure 2c,d shows SEM images of the zeolites impregnated with transition metals Cu and Ni, respectively. The presence of white dots on the surface is noted, which indicates the presence of metals in the zeolite, and the surfaces become more regular compared to NZ and H2NZ, which may be due to the level of dispersion and uniform distribution of the metals on the surface of the zeolite. Similar observations were reported by Trisunaryanti et al. [58].

#### 3.2.3. Textural Properties

Textural properties of natural and modified zeolite samples are listed in Table 5. Surface areas (SBET) were calculated by applying the BET adsorption model to nitrogen adsorption data. The results indicate that an ion exchange process with NH_4_^+^ decreased the surface area without significant Si/Al ratio changes. These results are consistent with those published by Alejandro et al. [59]. This decrease in the surface area of Cu5H2NZ and Ni5H2NZ can be attributed to the incorporation of metal ions onto the surface of zeolites or inside the pores of the zeolite structure. This incorporation of transition metals could potentially block the zeolite pores, as indicated by Veses et al. [60].

Furthermore, the average pore size decreased for this catalyst, implying that more micropores were created in the Cu and Ni impregnation [20]. Metal impregnation (Cu, Ni) onto the zeolite framework decreased its surface area. These phenomena may be caused by metal distribution on the catalyst surface. Some of the metal atoms may block the pore mouth of the zeolite [48]. Furthermore, it was observed that the total pore volume in Cu5H2NZ and Ni5H2NZ was lower compared to the NZ. This decrease in pore volume due to metal loading suggests pore blockage, which can be attributed to the effective deposition of metals on the zeolite surface or their potential presence within the zeolite pores.

#### 3.2.4. Thermal Analysis

**Figure 3:**
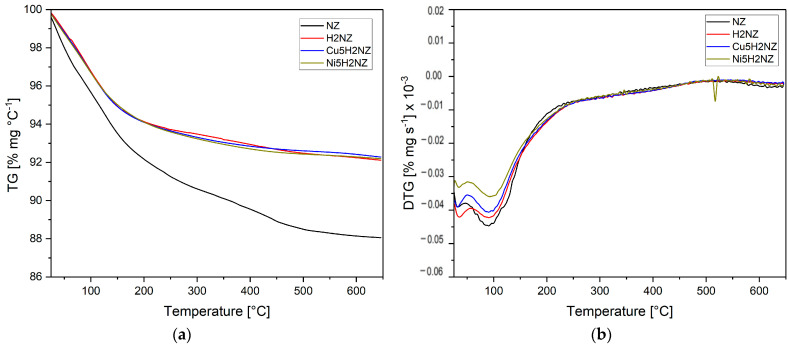
Thermal analysis of zeolite samples (**a**) TG and (**b**) DTG.

Natural zeolites impregnated with Cu and Ni must be thermally and chemically stable to be considered as potential catalysts. Figure 3 shows the results of the TG tests of the natural and modified zeolite samples after controlled heating up to 650 °C, at a rate of 10 °C∙min^−1^. The most significant weight loss (12%) is observed in the natural zeolite (NZ) sample. These results indicate that natural and modified zeolites are thermally stable, allowing their use in catalytic applications, as stated by Rajić et al. [23]. Weight loss was continuous during heating up to 650 °C. The lost weight of the zeolite is a result of the heat treatment due to dehydroxylation and dehydration of the surface. The higher weight loss occurs at the temperature range (20 to 350 °C), according to Perraki and Orfanoudaki [61], corresponding to hygroscopic water (up to 100 °C) and loosely bound water (100 to 200 °C), having a moderate loss in the 350 to 650 °C range, which could be mainly due to the elimination of the structural water (hydroxyl groups) of clinoptilolite. These results are similar to those obtained by Sprynskyy et al. [62].

#### 3.2.5. Acid Site Strength

**Figure 4:**
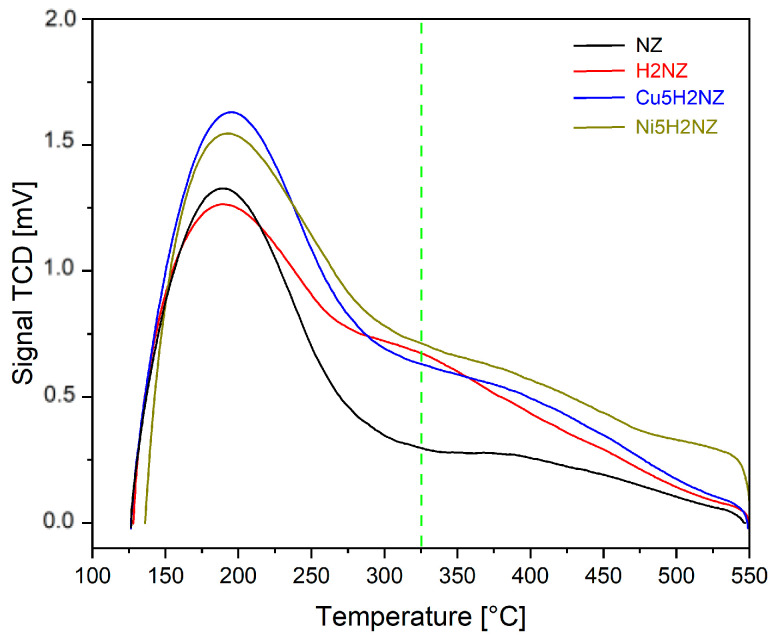
NH_3_-TPD for natural and modified zeolite samples.

NH_3_-TPD characterizes the strength of accessible acid sites in the catalysts, as Figure 4 represents. 

The NH_3_ desorption occurs across a wide temperature range, indicating weak and strong acid sites in all the samples. The peaks with a maximum below 325 °C correspond to weak acidic sites, as those above 325 °C represent strong acidic sites [63]. An increase in the total acidic sites was observed for impregnated zeolites. It can be associated with metals (Cu and Ni) that are highly coordinated (they can bond with the ligand due to the arrangement of the electrons in the unoccupied d orbital), which has low-energy orbitals and can act as a weak acidic site [64].

Table 6 shows the strength of the acid sites in the natural and modified zeolite samples. A high number and density of acid sites are desirable for deoxygenation reactions and the production of aromatic compounds during the pyrolysis of lignocellulosic biomass [65]. H2NZ posed stronger acid sites than NZ, promoted by Brönsted acid sites associated with hydroxyl groups, generated after ion exchange with NH_4_^+^ and thermal treatment [12]. Impregnation introduced Cu^2+^ and Ni^2+^ cations, present as CuO and NiO oxides, as confirmed in the XRD analysis. The presence of these oxides led to an increase in strong acid sites at lower temperatures, which aligns with what was published by Veses et al. [22]. 

### 3.3. Catalytic Fast Pyrolysis of Biomass

#### 3.3.1. Family Compounds Formed during PR and EG Catalytic Pyrolysis

A GC/MS analysis was employed to identify obtained compounds during the catalytic pyrolysis of PR and EG using NZ, H2NZ, Cu5H2NZ, and Ni5H2NZ as catalysts for the biomass–catalyst combinations: 1/1, 1/2.5, and 1/5. The family compounds detected are shown in Table S1 of the Supplementary Information. The compounds and compound families were quantified using a semi-quantitative approach that determined the relative area percentage of all identified constituents, which were categorized into different groups (phenols, acids, aldehydes, furans, ketones, aromatics, and esters) based on previous reports [66]. Non-catalytic pyrolysis assays were also conducted to compare catalyst performance. 

Oxygenated compounds, such as acids, aldehydes, esters, and ketones, are considered undesirable fractions for energy production, while hydrocarbons and alcohols are regarded as desirable products for biofuel production. Phenols and furans are also seen as high-value chemicals [67]. The data indicate that non-catalytic pyrolysis led to the production of condensate vapors with a high content of oxygenated compounds, primarily yielding approximately 5.40% aldehydes, 8.74% acids, 12.09% esters, and 18.75% ketones in PR and 14.51% aldehydes, 15.31% acids, 6.37% esters, and 13.78% ketones in EG. Other oxygenated compounds were also present, including 5.72% alcohols, 4.62% furans, and 32.77% phenols in PR. On the other hand, 4.61% alcohols, 4.94% furans, and 24.06% phenols were observed when EG was used. This higher proportion of phenols was expected to result primarily from the pyrolysis of the lignin component of the biomass. At the same time, the production of acids and ketones could be chiefly attributed to the thermal degradation of cellulose, hemicellulose, and some portion of lignin [68].

Further analysis using catalyst samples (NZ, H2NZ, Cu5H2NZ, and Ni5H2NZ) revealed a significant reduction in the formation of acids, esters, ketones, alcohols, and phenols [69]. There was a pronounced reduction of ketones for PR and less evident for EG. Likewise, Cu5H2NZ samples promoted 22% phenols and 10% ketones, and Ni5H2NZ 27.5% phenols and 12.5% ketones in EG pyrolysis. Such results suggest that the catalysts favored deoxygenation pathways such as dehydration, decarboxylation, and decarbonylation to convert oxygenated compounds [70]. The presence of acidic compounds can be attributed to cellulose and hemicellulose depolymerization due to dehydration, C-C bond cleavage, and direct C-O bond cleavage occurring at the acidic sites of catalysts [68]. 

Thus, the increased deoxygenation activity of Ni5H2NZ, compared to Cu5H2NZ, can be attributed to its higher surface area, porosity, and acid site density. A predominant deoxygenation reaction for the Cu5H2NZ sample could be possibly associated with acid decarbonylation to form aldehydes at copper cations. On the other hand, the preferred mechanism for the O-removal using Ni5H2NZ samples seems to involve decarbonylation and decarboxylation reactions occurring at the acid sites formed after metal integration. Although a dominant reaction mechanism could not be assured, the lower incorporation of cations at ion exchange positions could potentially assess the reduced deoxygenation rate [60]. Additionally, they demonstrated that the increased aromatics fraction correlates with the increased number of accessible acid sites at the mesopore surface, where preferential occurrence of decarbonylation reactions is essential for enhancing aromatic production. The deoxygenation of condensed vapors increases with the number of weak acid sites created by incorporating cations on the external surface and mesoporous walls, confirming previously reported results by Veses et al. [60], who demonstrated a direct relationship between the deoxygenation rate and the weak acid sites increment [11].

The higher production of aromatic compounds was reported for EG (around 30%), while in PR, aromatics were close to 20%. Additionally, aromatics production is higher at a B/C ratio of 1/5 in PR and 1/2.5 in EG for all catalysts. The deoxygenation and increased proportion of aromatics are related to a higher catalyst amount, allowing more active sites to interact with pyrolytic vapors, resulting in greater deoxygenation. 

**Figure 5:**
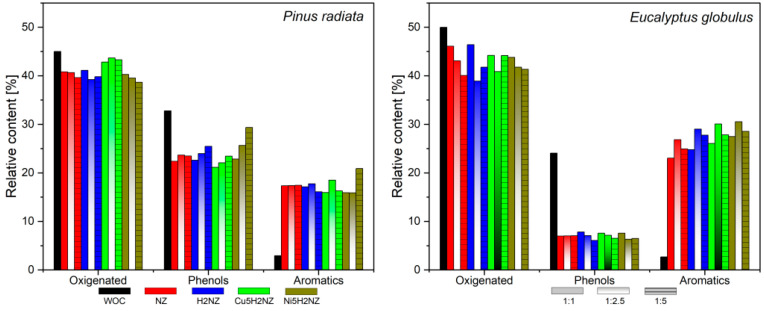
Family compounds from biomass catalytic pyrolysis.

Figure 5 displays the compound families formed during biomass pyrolysis and catalytic biomass pyrolysis. The single color bars correspond to the 1/1 biomass/catalyst, the bars with two colors (colored and white) correspond to 1/2.5, and the bars’ horizontal line corresponds to the 1/5 condition. The black bars correspond to the trials without a catalyst (WOC), the red bars correspond to NZ, the blue bars to H2NZ, the green bars to Cu5H2NZ, and the brown bars to Ni5H2NZ. The components detected in the condensed vapors were divided into three groups: oxygenates, phenols, and aromatics. Aromatics were divided into monocyclic aromatic hydrocarbons (MAHs), mainly benzene, toluene, and xylene (BTX), and polycyclic aromatic hydrocarbons (PAHs). Moreover, oxygenate production was based on alcohols, aldehydes, carboxylic acids, esters, furans, and ketones.

#### 3.3.2. Reaction Pathways

In biomass catalytic pyrolysis, similar mechanisms enhanced the conversion of oxygenated compounds (acids, ketones, esters, phenols, aldehydes, among others) obtained through the thermochemical transformation of cellulose, hemicellulose, and lignin [22]. The conversion of catalytic pyrolysis vapors occurs through dehydration, decarbonylation, decarboxylation, aromatization, and condensation reactions on zeolite active sites [71]. 

**Figure 6:**
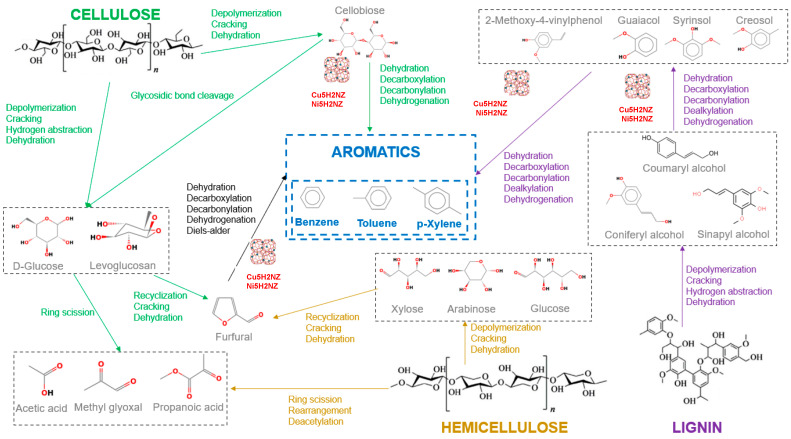
Proposed reactions scheme. Adapted from [28,77,78,79].

Figure 6 shows a potential reaction pathway of PR and EG catalytic pyrolysis using NZ, H2NZ, Cu5H2NZ, and Ni5H2NZ, considering intermediate products such as oxygenates, furans, phenols, and acids [72]. Such compound families reach the so-called “hydrocarbon pool”, where alcohols, ketones, and aldehydes are produced through deoxygenation, decarbonization, and oligomerization reactions. Subsequently, aromatic compounds are formed through aromatization, dehydration, decarbonization, and polymerization reactions [73].

The analytical results indicate that the presence of catalysts (NZ, H2NZ, Cu5 H2NZ, and Ni5H2NZ) can enhance aromatics production from biomass catalytic conversion. Ni5H2NZ can better promote the formation of BTX and reduce the formation of oxygenated compounds than Cu5H2NZ.

During biomass catalytic pyrolysis, oxygenated pyrolytic vapors with large molecules were first broken down into smaller hydrocarbon precursors (including monofunctional furans, ketones, and light phenols) by the metallic oxides, which favored both diffusion and catalytic reactions in the zeolite, resulting in the improved formation of aromatic compounds [74].

The decomposition of cellulose and hemicellulose generates oxygenated compounds, including anhydrosugars, acids, and furfural. These compounds undergo decarboxylation, ketonization, and aldol condensation reactions over the metallic oxides to produce furans and ketones, which are then transformed into aromatic compounds [75]. Furans are indispensable intermediates for the generation of BTX, which the catalyst promotes.

The “hydrocarbon pool”, a mass of olefins, is produced through oligomerization and can be converted into aromatics via aromatization over the catalyst. The generated olefins can also react with different furans to form aromatics through the Diels–Alder reaction [8]. 

Ketones also undergo aromatization in the catalyst channels through self-condensation, decarboxylation, and decarbonylation reactions. Moreover, light oxygenates could be deoxygenated and cracked to C2 to C6 olefins over the catalyst, which would be aromatized into benzene and other aromatics [76].

#### 3.3.3. Aromatics Compounds

The highest production of aromatic compounds was observed for *Pinus radiata* (20.89% with Ni5H2NZ, biomass/catalysts = 1/5) and *Eucalyptus globulus* (35.53% with Cu5H2NZ, biomass/catalysts = 1/2.5), with a generally higher production of aromatics in the catalytic pyrolysis of EG than PR. Then, aromatics production was at its maximum at a B/C ratio of 1/2.5 for PR and 1/5 for EG. Deoxygenation and aromatics increments are related to a higher density of active sites in the catalysts interacting with pyrolytic vapors. Additionally, the increase in the aromatic fraction could be related to more available strong acid sites on the catalysts’ surface, which are responsible for decarbonylation reactions and essential for improving aromatic production. Deoxygenation increased with the density of weak acid sites formed by incorporating cations on the external surface and mesoporous walls, as reported elsewhere [11,60].

During catalytic pyrolysis, a biomass/catalyst ratio of 2.5 proved the most significant in achieving maximum aromatic formation. A higher amount of catalyst contributes to more active sites involved with pyrolytic vapors, resulting in deoxygenation of condensable vapors and enhanced aromatic production compared to biomass/catalyst ratios of 1/1 and 1/5. All catalysts showed a reduction in the proportion of oxygenated compounds. Adding Cu or Ni also improved the efficiency of the catalysts in deoxygenating condensable vapors. The proportion of acidic compounds was considerably reduced. These results align with those published by Tian et al. [44].

This efficient aromatic production can be attributed to the excellent catalytic activity of Cu and Ni cations and physicochemical properties such as higher surface area and an increased number of acidic sites. Additionally, this production can be attributed to the aromatization activity carried out by the acidic sites present in the catalysts and other deoxygenation pathways, such as dehydration, decarboxylation, and decarbonylation, which were efficiently catalyzed by the catalysts [8].

Table S2 shows the effect of the catalysts on BTX yields, and Table S3 presents the effect of the catalysts on the aromatic’s selectivity. The total BTX selectivity increased after adding the catalysts. These yields obtained with (NZ, H2NZ, Cu5H2NZ, and Ni5H2NZ) were attributed to their suitable pore structure and acidic strength. Aromatics were derived from the selective catalytic fast pyrolysis of oxygenates (especially G and S-type phenols) through dihydroxylation and demethoxylation reactions [80]. These intermediate oxygenates entered the zeolite channels and underwent a series of deoxygenation reactions at the acid sites [43]. Among the BTX compounds, toluene was the most prevalent for *Pinus radiata* and *Eucalyptus globulus*. Thus, the selective yields of toluene and xylene in the 1/2.5 ratio were higher than those of benzene because toluene and xylene can be directly produced from the dehydration (-OH) and demethoxylation (-OCH_3_) of oxygenated precursors (especially phenolics). In contrast, an additional step is required to produce benzene compared to the steps needed for toluene and xylene: the demethylation (-CH_3_) of toluene and xylene [81]. Therefore, obtaining benzene is more challenging than toluene and xylene, hence the lower yield [80].

## 4. Conclusions

This study evaluated the influence of metal-supported Chilean natural zeolites in the analytical pyrolysis of *Pinus radiata* and *Eucalyptus globulus*. Natural zeolite was modified to incorporate transition metals (Cu and Ni) on the surface through incipient wet impregnation. SEM-EDS analyses confirmed the successful impregnation (6.20% for Cu5H2NZ and 6.97% for Ni5H2NZ). XRD analysis showed the zeolite framework’s incorporated metal oxides (CuO and NiO).

The oxygenated compounds reduction, such as acids, esters, ketones, and phenols, was observed after using Py-GC/MS and comparing biomass non-catalytic pyrolysis (of PR and EG) with catalytic pyrolysis using ZN, H2ZN, Cu5H2NZ, and Ni5H2NZ. Along with the reduction in oxygenated compounds, an increase in aromatic compounds was confirmed in PR from 2.92% *w*/*w* to 20.89% *w*/*w* with Ni5H2NZ and a biomass/catalyst ratio of 1/5, and in EG from 2.69% *w*/*w* to 30.53% *w*/*w* with Ni5H2NZ and a biomass/catalyst ratio of 1/2.5, demonstrating the direct contribution of the modified catalyst toward aromatic compounds selectivity.

Deoxygenation and the increment in aromatics are related to a higher density of active sites in the catalysts interacting with pyrolytic vapors. Additionally, the increase in the aromatic fraction could be related to more available strong acid sites on the catalysts’ surface, responsible for decarbonylation reactions and essential for improving aromatic production. Deoxygenation increased with the density of weak acid sites formed by incorporating cations on the external surface and mesoporous walls. The higher deoxygenation activity and aromatic compound production by the catalysts can be attributed to the combination of acidic and metallic sites within the pores or on the surface of the catalysts, facilitating significant deoxygenation reactions such as dehydration, decarboxylation, decarbonylation, aldol condensation, and aromatization.

Finally, this study demonstrated that catalytic biomass pyrolysis using metal-impregnated (Cu and Ni) zeolites could significantly convert oxygenated compounds into hydrocarbons and improve the quality of condensed pyrolysis vapors.

## Figures and Tables

**Table 1:** Pyrolysis conditions.

Sample	Plots	Ref		
Catalysts	NZ	H2NZ	Cu5H2NZ	Ni5H2NZ
Relation B/C	1/1	1/2.5	1/5	
Biomass	PR	EG		

**Table 2:** Characterization results of biomass samples.

	Proximate Analysis			Ultimate Analysis			Chemical Composition	
	PR	EG		PR	EG		PR	EG
Moisture (%)	7.75	5.51	Carbon (%)	48.02	47.76	Hemicellulose (%)	28.6 ± 0.8	26.3 ± 0.3
Volatiles (%)	76.73	77.13	Hydrogen (%)	5.90	6.32	Cellulose (%)	43.1 ± 0.1	53.0 ± 0.2
Fixed carbon (%)	14.68	16.85	Nitrogen (%)	0.29	0.09	Lignin (%)	26.6 ± 1.6	23.9 ± 2.1
Ash (%)	0.83	0.51	Sulphur (%)	0.10	0.05	Extractives (%)	1.8 ± 0.3	1.9 ± 0.0
			Oxygen (%)	45.69	45.77			

**Table 3:** Crystal size in zeolite samples.

Sample	D (nm)	Crystallinity
NZ		98.38
H2NZ		100.00
Cu5H2NZ	7.25	95.77
Ni5H2NZ	9.49	94.38

**Table 4:** Physico-chemical characterization of the Chilean natural zeolite and impregnated.

Sample	Al	Si	Na	K	Mg	Ca	Ti	Mn	Fe	Cu	Ni	Si/Al
NZ	6.43	31.09	0.89	0.87	0.57	1.82	0.26	0.04	2.31	nd	nd	4.83
H2NZ	6.45	31.90	0.49	0.82	0.54	1.12	0.26	0.04	2.33	nd	nd	4.95
Cu5H2NZ	6.25	32.58	nd	0.80	0.36	1.00	0.22	0.03	2.34	4.66	nd	5.21
Ni5H2NZ	5.13	27.76	0.38	0.73	0.61	0.97	0.21	0.05	2.37	nd	5.93	5.42

nd: Not detected.

**Table 5:** Textural properties of catalysts.

Sample	S_BET_ (m^2^·g^−1^)	Pore Size (nm)	V_micropore_ (cm^3^·g^−1^)	V_mesopore_ (cm^3^·g^−1^)
NZ	160.59	1.77	0.063	0.13
H2NHZ	111.73	1.71	0.042	0.11
Cu5H2NZ	99.34	1.55	0.047	0.10
Ni5H2NZ	132.30	1.55	0.052	0.10

**Table 6:** Physico-chemical characterization of the natural and impregnated zeolites.

	Area [μmol·g^−1^]			Peak Temperature [°C]	
Sample	Weak Sites	Strong Sites	Total Sites	Weak Sites	Strong Sites
NZ	148.42	50.40	198.82	190.1	382.6
H2NZ	165.48	98.59	264.07	193.2	338.8
Cu5H2NZ	197.82	92.54	290.30	194.0	390.3
Ni5H2NZ	190.51	124.80	315.31	195.3	397.2

## Data Availability

The original contributions presented in the study are included in the article/Supplementary Material, further inquiries can be directed to the corresponding author.

## Supplementary Materials

The following supporting information can be downloaded at: https://www.mdpi.com/article/10.3390/polym16131912/s1, Table S1. Family compounds of catalytic pyrolysis of biomass. Table S2. BTX yields. Table S3. BTX selectivity.

## References

[1] BP (2021). Full Report—Statistical Review of World Energy 2021.

[2] Deng W., Feng Y., Fu J., Guo H., Guo Y., Han B., Jiang Z., Kong L., Li C., Liu H. (2023). Catalytic Conversion of Lignocellulosic Biomass into Chemicals and Fuels. Green Energy Environ..

[3] Hu X., Gholizadeh M. (2019). Biomass Pyrolysis: A Review of the Process Development and Challenges from Initial Researches up to the Commercialisation Stage. J. Energy Chem..

[4] Álvarez González V., Poblete Hernández P., Soto Aguirre D., Caselli J.G., Kahler González C., Pardo Velásquez E., Carlos J., Munita B., Rocha D.B. (2022). Anuario Forestal Chilean Statistical Yearbook of Forestry 2022. Anu. For. INFOR.

[5] Arteaga-Pérez L., Segura C., Diéguez K. (2016). Procesos de Torrefacción Para Valorización de Residuos Lignocelulósicos. Análisis de Posibles Tecnologías de Aplicación En Sudamérica. Afinidad.

[6] Xiu S., Shahbazi A. (2012). Bio-Oil Production and Upgrading Research: A Review. Renew. Sustain. Energy Rev..

[7] Chaihad N., Karnjanakom S., Abudula A., Guan G. (2022). Zeolite-Based Cracking Catalysts for Bio-Oil Upgrading: A Critical Review. Resour. Chem. Mater..

[8] Kumar R., Strezov V., Lovell E., Kan T., Weldekidan H., He J., Jahan S., Dastjerdi B., Scott J. (2019). Enhanced Bio-Oil Deoxygenation Activity by Cu/Zeolite and Ni/Zeolite Catalysts in Combined in-Situ and Ex-Situ Biomass Pyrolysis. J. Anal. Appl. Pyrolysis.

[9] Kan T., Strezov V., Evans T.J. (2016). Lignocellulosic Biomass Pyrolysis: A Review of Product Properties and Effects of Pyrolysis Parameters. Renew. Sustain. Energy Rev..

[10] Mohan D., Pittman C.U., Steele P.H. (2006). Pyrolysis of Wood/Biomass for Bio-Oil: A Critical Review. Energy Fuels.

[11] Huo X., Xiao J., Song M., Zhu L. (2018). Comparison between In-Situ and Ex-Situ Catalytic Pyrolysis of Sawdust for Gas Production. J. Anal. Appl. Pyrolysis.

[12] Galadima A., Muraza O. (2015). In Situ Fast Pyrolysis of Biomass with Zeolite Catalysts for Bioaromatics/Gasoline Production: A Review. Energy Convers. Manag..

[13] Wang K., Johnston P.A., Brown R.C. (2015). Comparison of In-Situ and Ex-Situ Catalytic Pyrolysis in a Micro-Reactor System. Bioresour. Technol..

[14] Liu R., Sarker M., Rahman M.M., Li C., Chai M., Nishu, Cotillon R., Scott N.R. (2020). Multi-Scale Complexities of Solid Acid Catalysts in the Catalytic Fast Pyrolysis of Biomass for Bio-Oil Production—A Review. Prog. Energy Combust. Sci..

[15] Barbosa A.S., Siqueira L.A.M., Medeiros R.L.B.A., Melo D.M.A., Melo M.A.F., Freitas J.C.O., Braga R.M. (2019). Renewable Aromatics through Catalytic Flash Pyrolysis of Pineapple Crown Leaves Using HZSM-5 Synthesised with RHA and Diatomite. Waste Manag..

[16] Alejandro-Martín S., Acaricia A.M., Cerda-Barrera C., Pérez H.D. (2019). Influence of Chemical Surface Characteristics of Ammonium-Modified Chilean Zeolite on Oak Catalytic Pyrolysis. Catalysts.

[17] Dehmani Y., Ba Mohammed B., Oukhrib R., Dehbi A., Lamhasni T., Brahmi Y., El-Kordy A., Franco D.S.P., Georgin J., Lima E.C. (2024). Adsorption of Various Inorganic and Organic Pollutants by Natural and Synthetic Zeolites: A Critical Review. Arab. J. Chem..

[18] Shekarchi M., Ahmadi B., Azarhomayun F., Shafei B., Kioumarsi M. (2023). Natural Zeolite as a Supplementary Cementitious Material—A Holistic Review of Main Properties and Applications. Constr. Build. Mater..

[19] Serrano D.P., Melero J.A., Morales G., Iglesias J., Pizarro P. (2018). Progress in the Design of Zeolite Catalysts for Biomass Conversion into Biofuels and Bio-Based Chemicals. Catal. Rev. Sci. Eng..

[20] Gurevich Messina L.I., Bonelli P.R., Cukierman A.L. (2017). In-Situ Catalytic Pyrolysis of Peanut Shells Using Modified Natural Zeolite. Fuel Process. Technol..

[21] Bhoi P.R., Ouedraogo A.S., Soloiu V., Quirino R. (2020). Recent Advances on Catalysts for Improving Hydrocarbon Compounds in Bio-Oil of Biomass Catalytic Pyrolysis. Renew. Sustain. Energy Rev..

[22] Veses A., Puértolas B., Callén M.S., García T. (2015). Catalytic Upgrading of Biomass Derived Pyrolysis Vapors over Metal-Loaded ZSM-5 Zeolites: Effect of Different Metal Cations on the Bio-Oil Final Properties. Microporous Mesoporous Mater..

[23] Rajić N., Logar N.Z., Rečnik A., El-Roz M., Thibault-Starzyk F., Sprenger P., Hannevold L., Andersen A., Stöcker M. (2013). Hardwood Lignin Pyrolysis in the Presence of Nano-Oxide Particles Embedded onto Natural Clinoptilolite. Microporous Mesoporous Mater..

[24] Cruz N., Bustos C., Aguayo M.G., Cloutier A., Castillo R. (2018). THM Densification of Wood. Bioresources.

[25] Alejandro S., Valdés H., Manero M.H., Zaror C.A. (2012). BTX Abatement Using Chilean Natural Zeolite: The Role of Brønsted Acid Sites. Water Sci. Technol..

[26] Alejandro-Martín S., Valdés H., Zaror C.A. (2011). Natural Zeolite Reactivity towards Ozone: The Role of Acid Surface Sites. J. Adv. Oxid. Technol..

[27] Soled S.L., Malek A., Miseo S., Baumgartner J., Kliewer C., Afeworki M., Stevens P.A. (2006). Supported Metal Catalysts: Some Interesting New Leads in an Old Field. Studies in Surface Science and Catalysis.

[28] Chaihad N., Anniwaer A., Karnjanakom S., Kasai Y., Kongparakul S., Samart C., Reubroycharoen P., Abudula A., Guan G. (2021). In-Situ Catalytic Upgrading of Bio-Oil Derived from Fast Pyrolysis of Sunflower Stalk to Aromatic Hydrocarbons over Bifunctional Cu-Loaded HZSM-5. J. Anal. Appl. Pyrolysis.

[29] Persson H., Duman I., Wang S., Pettersson L.J., Yang W. (2019). Catalytic Pyrolysis over Transition Metal-Modified Zeolites: A Comparative Study between Catalyst Activity and Deactivation. J. Anal. Appl. Pyrolysis.

[30] (2017). Standard Test Methods for Instrumental Determination of Carbon, Hydrogen, and Nitrogen in Laboratory Samples of Coal.

[31] (2013). Standard Practice for Proximate Analysis of Coal and Coke.

[32] (2015). Acetone Extractives of Wood and Pulp.

[33] Aguayo M.G., Quintupill L., Castillo R., Baeza J., Freer J., Mendonça R.T. (2010). Determination of Differences in Anatomical and Chemical Characteristics of Tension and Opposite Wood of 8-Year Old Eucalyptus Globulus. Maderas Cienc. Y Tecnol..

[34] Venegas-Vásconez D., Arteaga-Pérez L.E., Aguayo M.G., Romero-Carrillo R., Guerrero V.H., Tipanluisa-Sarchi L., Alejandro-Martín S. (2023). Analytical Pyrolysis of Pinus Radiata and Eucalyptus Globulus: Effects of Microwave Pretreatment on Pyrolytic Vapours Composition. Polymers.

[35] Sihombing J.L., Gea S., Wirjosentono B., Agusnar H., Pulungan A.N., Herlinawati H., Yusuf M. (2020). Characteristic and Catalytic Performance of Co and Co-Mo Metal Impregnated in Sarulla Natural Zeolite Catalyst for Hydrocracking of MEFA Rubber Seed Oil into Biogasoline Fraction. Catalysts.

[36] (2019). Standard Test Method for Determination of Relative Crystallinity of Zeolite Sodium A by X-ray Diffraction.

[37] Galarza E.D., Fermanelli C.S., Pierella L.B., Saux C., Renzini M.S. (2021). Influence of the Sn Incorporation Method in ZSM-11 Zeolites in the Distribution of Bio-Oil Products Obtained from Biomass Pyrolysis. J. Anal. Appl. Pyrolysis.

[38] Zhang C., Kwak G., Park H.G., Jun K.W., Lee Y.J., Kang S.C., Kim S. (2019). Light Hydrocarbons to BTEX Aromatics over Hierarchical HZSM-5: Effects of Alkali Treatment on Catalytic Performance. Microporous Mesoporous Mater..

[39] Alejandro-Martín S. (2013). Estudio de La Reacción Ozono-Compuestos Orgánicos Volátiles a Temperatura Ambiente En Presencia de Zeolita Natural Modificada. Ph.D. Thesis.

[40] Valdés H., Solar V.A., Cabrera E.H., Veloso A.F., Zaror C.A. (2014). Control of Released Volatile Organic Compounds from Industrial Facilities Using Natural and Acid-Treated Mordenites: The Role of Acidic Surface Sites on the Adsorption Mechanism. Chem. Eng. J..

[41] Yogalakshmi K.N., Poornima Devi T., Sivashanmugam P., Kavitha S., Yukesh Kannah R., Varjani S., AdishKumar S., Kumar G., Rajesh Banu J. (2022). Lignocellulosic Biomass-Based Pyrolysis: A Comprehensive Review. Chemosphere.

[42] Hubble A.H., Childs B.A., Pecchi M., Sudibyo H., Tester J.W., Goldfarb J.L. (2023). Role of in Situ (in Contact with Biomass) and Ex Situ (in Contact with Pyrolysis Vapors) Transition Metal Catalysts on Pyrolysis of Cherry Pits. Fuel.

[43] Huang M., Xu J., Ma Z., Yang Y., Zhou B., Wu C., Ye J., Zhao C., Liu X., Chen D. (2021). Bio-BTX Production from the Shape Selective Catalytic Fast Pyrolysis of Lignin Using Different Zeolite Catalysts: Relevance between the Chemical Structure and the Yield of Bio-BTX. Fuel Process. Technol..

[44] Tian H., Chen L., Huang Z., Cheng S., Yang Y. (2022). Increasing the Bio-Aromatics Yield in the Biomass Pyrolysis Oils by the Integration of Torrefaction Deoxygenation Pretreatment and Catalytic Fast Pyrolysis with a Dual Catalyst System. Renew. Energy.

[45] Niu Q., Ghysels S., Wu N., Rousseau D.P.L., Pieters J., Prins W., Ronsse F. (2022). Effects of Demineralization on the Composition of Microalgae Pyrolysis Volatiles in Py-GC–MS. Energy Convers. Manag..

[46] Arteaga-Pérez L.E., Segura C., Espinoza D., Radovic L.R., Jiménez R. (2015). Torrefaction of Pinus Radiata and Eucalyptus Globulus: A Combined Experimental and Modeling Approach to Process Synthesis. Energy Sustain. Dev..

[47] Wang Y., Akbarzadeh A., Chong L., Du J., Tahir N., Awasthi M.K. (2022). Catalytic Pyrolysis of Lignocellulosic Biomass for Bio-Oil Production: A Review. Chemosphere.

[48] Trisunaryanti W. (2013). Characteristics of Metal Supported-Zeolite Catalysts for Hydrocracking of Polyethylene Terephthalat. IOSR J. Appl. Chem..

[49] Kadarwati S., Wahyuni S., Trisunaryanti W., Triyono T. (2010). Preparation, Characterizacion and Catalytic Activity Test of Ni-Mo/Natural Zeolite on Pyridine Hydrodenitrogenation. Indones. J. Chem..

[50] Zhang J., Huang Y., Sekyere D.T., Wang W., Tian Y. (2024). Catalytic Fast Pyrolysis of Waste Pine Sawdust over Solid Base, Acid and Base-Acid Tandem Catalysts. Bioresour. Technol..

[51] Zheng Y., Wang F., Yang X., Huang Y., Liu C., Zheng Z., Gu J. (2017). Study on Aromatics Production via the Catalytic Pyrolysis Vapor Upgrading of Biomass Using Metal-Loaded Modified H-ZSM-5. J. Anal. Appl. Pyrolysis.

[52] Seyed Mousavi S.A.H., Sadrameli S.M., Saeedi Dehaghani A.H. (2022). Catalytic Pyrolysis of Municipal Plastic Waste over Nano MIL-53 (Cu) Derived @ Zeolite Y for Gasoline, Jet Fuel, and Diesel Range Fuel Production. Process Saf. Environ. Prot..

[53] Alshameri A., Ibrahim A., Assabri A.M., Lei X., Wang H., Yan C. (2014). The Investigation into the Ammonium Removal Performance of Yemeni Natural Zeolite: Modification, Ion Exchange Mechanism, and Thermodynamics. Powder Technol..

[54] Pabalan R.T., Bertetti F.P. (2001). Cation-Exchange Properties of Natural Zeolites. Rev. Mineral. Geochem..

[55] Ates A., Hardacre C. (2012). The Effect of Various Treatment Conditions on Natural Zeolites: Ion Exchange, Acidic, Thermal and Steam Treatments. J. Colloid Interface Sci..

[56] Ünaldi T., Mizrak I., Kadir S. (2013). Physicochemical Characterisation of Natural K-Clinoptilolite and Heavy-Metal Forms from Gördes (Manisa, Western Turkey). J. Mol. Struct..

[57] Alejandro S., Valdés H., Manéro M.H., Zaror C.A. (2014). Oxidative Regeneration of Toluene-Saturated Natural Zeolite by Gaseous Ozone: The Influence of Zeolite Chemical Surface Characteristics. J. Hazard Mater..

[58] Trisunaryanti W., Triyono, Falah I.I., Widyawati D., Yusniyanti F. (2024). The Effect of Oxalic Acid and NaOH Treatments on the Character of Wonosari Natural Zeolite as Ni, Cu, and Zn Metal Support Catalyst for Hydrocracking of Castor Oil. Biomass Convers. Biorefinery.

[59] Alejandro-Martín S., Valdés H., Manero M.H., Zaror C.A. (2018). Catalytic Ozonation of Toluene Using Chilean Natural Zeolite: The Key Role of Brønsted and Lewis Acid Sites. Catalysts.

[60] Veses A., Puértolas B., López J.M., Callén M.S., Solsona B., García T. (2016). Promoting Deoxygenation of Bio-Oil by Metal-Loaded Hierarchical ZSM-5 Zeolites. ACS Sustain. Chem. Eng..

[61] Perraki T., Orfanoudaki A. (2004). Mineralogical Study of Zeolites from Pentalofos Area, Thrace, Greece. Appl. Clay Sci..

[62] Sprynskyy M., Golembiewski R., Trykowski G., Buszewski B. (2010). Heterogeneity and Hierarchy of Clinoptilolite Porosity. J. Phys. Chem. Solids.

[63] Li Y., Liu S., Xie S., Xu L. (2009). Promoted Metal Utilization Capacity of Alkali-Treated Zeolite: Preparation of Zn/ZSM-5 and Its Application in 1-Hexene Aromatization. Appl. Catal. A Gen..

[64] Wibowo S., Efiyanti L., Pari G. (2020). Catalytic and Thermal Cracking of Bio-Oil from Oil-Palm Empty Fruit Bunches, in Batch Reactor. Indones. J. Chem..

[65] Valizadeh S., Pyo S., Kim Y., Hakimian H., Park Y. (2022). Production of Aromatics Fuel Additives from Catalytic Pyrolysis of Cow Manure over HZSM-5, HBeta, and HY Zeolites. Chem. Eng. J..

[66] Wang H., Male J., Wang Y. (2013). Recent Advances in Hydrotreating of Pyrolysis Bio-Oil and Its Oxygen-Containing Model Compounds. ACS Catal..

[67] Iliopoulou E.F., Stefanidis S.D., Kalogiannis K.G., Delimitis A., Lappas A.A., Triantafyllidis K.S. (2012). Catalytic Upgrading of Biomass Pyrolysis Vapors Using Transition Metal-Modified ZSM-5 Zeolite. Appl. Catal. B.

[68] Yang H., Yan R., Chen H., Lee D.H., Zheng C. (2007). Characteristics of Hemicellulose, Cellulose and Lignin Pyrolysis. Fuel.

[69] Cheng Y.T., Huber G.W. (2011). Chemistry of Furan Conversion into Aromatics and Olefins over HZSM-5: A Model Biomass Conversion Reaction. ACS Catal..

[70] Zhang J., Fidalgo B., Kolios A., Shen D., Gu S. (2018). Mechanism of Deoxygenation in Anisole Decomposition over Single-Metal Loaded HZSM-5: Experimental Study. Chem. Eng. J..

[71] Iliopoulou E.F., Triantafyllidis K.S., Lappas A.A. (2019). Overview of Catalytic Upgrading of Biomass Pyrolysis Vapors toward the Production of Fuels and High-Value Chemicals. Wiley Interdiscip. Rev. Energy Environ..

[72] Chen W.H., Cheng C.L., Lee K.T., Lam S.S., Ong H.C., Ok Y.S., Saeidi S., Sharma A.K., Hsieh T.H. (2021). Catalytic Level Identification of ZSM-5 on Biomass Pyrolysis and Aromatic Hydrocarbon Formation. Chemosphere.

[73] Williams P.T., Horne P.A. (1995). The Influence of Catalyst Type on the Composition of Upgraded Biomass Pyrolysis Oils. J. Anal. Appl. Pyrolysis.

[74] Stefanidis S.D., Karakoulia S.A., Kalogiannis K.G., Iliopoulou E.F., Delimitis A., Yiannoulakis H., Zampetakis T., Lappas A.A., Triantafyllidis K.S. (2016). Natural Magnesium Oxide (MgO) Catalysts: A Cost-Effective Sustainable Alternative to Acid Zeolites for the in Situ Upgrading of Biomass Fast Pyrolysis Oil. Appl. Catal. B.

[75] Mante O.D., Rodriguez J.A., Senanayake S.D., Babu S.P. (2015). Catalytic Conversion of Biomass Pyrolysis Vapors into Hydrocarbon Fuel Precursors. Green Chem..

[76] Lazaridis P.A., Fotopoulos A.P., Karakoulia S.A., Triantafyllidis K.S. (2018). Catalytic Fast Pyrolysis of Kraft Lignin with Conventional, Mesoporous and Nanosized ZSM-5 Zeolite for the Production of Alkyl-Phenols and Aromatics. Front. Chem..

[77] Tawalbeh M., Al-Othman A., Salamah T., Alkasrawi M., Martis R., El-Rub Z.A. (2021). A Critical Review on Metal-Based Catalysts Used in the Pyrolysis of Lignocellulosic Biomass Materials. J. Environ. Manag..

[78] Chen X., Che Q., Li S., Liu Z., Yang H., Chen Y., Wang X., Shao J., Chen H. (2019). Recent Developments in Lignocellulosic Biomass Catalytic Fast Pyrolysis: Strategies for the Optimisation of Bio-Oil Quality and Yield. Fuel Process. Technol..

[79] Grams J., Jankowska A., Goscianska J. (2023). Advances in Design of Heterogeneous Catalysts for Pyrolysis of Lignocellulosic Biomass and Bio-Oil Upgrading. Microporous Mesoporous Mater..

[80] Alcazar-Ruiz A., Sanchez-Silva L., Dorado F. (2022). Enhancement of BTX Production via Catalytic Fast Pyrolysis of Almond Shell, Olive Pomace with Polyvinyl Chloride Mixtures. Process Saf. Environ. Prot..

[81] Huang M., Ma Z., Zhou B., Yang Y., Chen D. (2020). Enhancement of the Production of Bio-Aromatics from Renewable Lignin by Combined Approach of Torrefaction Deoxygenation Pretreatment and Shape Selective Catalytic Fast Pyrolysis Using Metal Modified Zeolites. Bioresour. Technol..

