# Supplementary material for: Biomass Valorization through Catalytic Pyrolysis Using Metal-Impregnated Natural Zeolites: From Waste to Resources
Source: Polymers (Basel). 2024 Jul 4;16(13):1912. doi: 10.3390/polym16131912 (PMC11244507; doi:10.3390/polym16131912)
Supplement: Supplementary file 1 [file polymers-16-01912-s001.zip › polymers-3027618-supplementary.pdf]

polymers

Article

Biomass Valorization through Catalytic Pyrolysis Using Metal-Impregnated Natural Zeolites: From Waste to Resources

Diego Venegas-Vásconez [1,2], Lourdes Orejuela-Escobar [3], Alfredo Valarezo-Garcés [4], Víctor H. Guerrero [5], Luis Tipanluisa-Sarchi [6] and Serguei Alejandro-Martín [1,2,*]

[1] Departamento de Ingeniería de Maderas, Universidad del Bío-Bío, Concepción 4081112, Chile; diego.venegas1801@alumnos.ubiobio.cl
[2] Laboratorio de Cromatografía Gaseosa y Pirólisis Analítica, Universidad del Bío-Bío, Concepción 4081112, Chile
[3] Departamento de Ingeniería Química, Universidad San Francisco de Quito USFQ, Diego de Robles s/n y Av. Interoceánica, Quito 170157, Ecuador; lorejuela@usfq.edu.ec
[4] Departamento de Ingeniería Mecánica, Universidad San Francisco de Quito USFQ, Diego de Robles s/n y Av. Interoceánica, Quito 170157, Ecuador; avalarezo@usfq.edu.ec
[5] Departamento de Materiales, Escuela Politécnica Nacional, Quito 170525, Ecuador; victor.guerrero@epn.edu.ec
[6] Facultad de Mecánica, Escuela Superior Politécnica de Chimborazo, Riobamba 060155, Ecuador; luis.tipanluisa@espoch.edu.ec
* Correspondence: salejandro@ubiobio.cl

polymers MDPI

Table S1. Catalytic pyrolysis compounds of PR and EG

		WOC	NZ			H2NZ			Cu5H2NZ			Ni5H2NZ		
		S/C	1:1	1:2.5	1:5	1:1	1:2.5	1:5	1:1	1:2.5	1:5	1:1	1:2.5	1:5
Acids	PR	8.75	5.76	6.39	6.42	5.68	5.90	7.15	6.65	7.42	7.41	3.30	5.23	5.95
	EG	15.31	13.58	12.61	11.64	14.19	13.10	11.64	14.11	13.36	13.58	13.17	13.06	12.07
Alcohols	PR	5.72	7.17	6.30	5.44	5.97	5.44	5.37	6.80	6.50	5.87	7.37	5.79	3.01
	EG	4.61	7.17	6.30	5.44	5.37	5.97	5.44	6.80	6.50	5.87	7.37	5.79	6.51
Aldehides	PR	5.40	18.59	17.73	16.74	18.98	17.17	16.35	18.78	18.62	18.52	19.77	18.00	15.37
	EG	14.51	12.79	12.95	13.11	16.57	13.63	14.69	12.99	11.21	12.95	12.52	13.38	12.20
Aromatics	PR	2.92	17.33	17.36	17.44	17.12	17.76	16.09	15.93	18.49	16.27	15.92	15.87	20.90
	EG	2.69	23.04	26.83	24.93	24.78	29.01	27.78	26.05	30.06	27.82	27.47	30.53	28.53
Cetons	PR	18.76	10.43	10.61	10.60	10.08	10.03	10.53	10.39	11.48	11.67	11.06	10.65	12.47
	EG	13.79	13.91	13.15	12.39	13.00	10.49	12.39	11.98	10.86	12.45	12.93	12.63	12.14
Esthers	PR	12.10	6.02	5.92	5.87	6.37	6.11	5.77	7.01	6.13	5.70	6.14	5.66	4.87
	EG	6.37	5.80	4.36	2.93	2.65	1.67	3.05	5.11	5.45	5.16	5.17	2.68	4.92
Furans	PR	4.63	7.43	8.16	9.39	7.96	8.05	9.61	7.11	7.86	9.05	6.34	6.57	7.98
	EG	4.95	8.38	10.11	11.84	9.18	8.28	10.15	8.33	7.26	8.81	8.24	8.42	8.89

Phenols	PR	32.77	22.38	23.66	23.48	22.59	23.97	25.46	21.14	22.06	23.43	22.84	25.64	29.35
	EG	24.06	6.98	7.01	7.04	7.81	7.07	6.09	7.57	7.13	6.52	7.56	6.33	6.49

Table S2. BTX yields

		NZ			H2NZ			Cu5H2NZ			Ni5H2NZ		
		1:1	1:2.5	1:5	1:1	1:2.5	1:5	1:1	1:2.5	1:5	1:1	1:2.5	1:5
PR	Benzene	2.05	3.13	3.44	3.21	3.41	3.29	3.15	3.53	3.23	2.62	2.60	3.35
	Toluene	7.33	7.76	7.26	7.33	7.43	7.14	8.68	7.88	8.15	8.21	6.90	8.51
	Xilene	3.21	3.82	3.83	4.04	3.94	3.59	3.60	4.10	3.57	3.37	3.25	4.25
EG	Benzene	0.55	0.21	1.94	1.24	2.47	2.16	1.32	2.44	2.17	1.41	1.79	2.39
	Toluene	7.09	6.84	8.29	8.06	8.34	8.29	8.50	8.39	8.62	7.92	7.67	8.09
	Xilene	6.41	6.20	6.72	7.21	7.13	6.72	7.21	7.03	7.08	7.57	7.51	7.83

Table S3. BTX selectivity

		NZ			H2NZ			Cu5H2NZ			Ni5H2NZ		
		1:1	1:2.5	1:5	1:1	1:2.5	1:5	1:1	1:2.5	1:5	1:1	1:2.5	1:5
PR	Benzene	18.55	18.01	19.71	18.78	19.18	20.46	19.79	19.11	19.86	21.14	20.47	20.35
	Toluene	42.31	44.72	41.65	42.83	41.83	44.38	54.51	42.62	50.06	51.57	43.46	40.74
	Xilene	11.83	21.99	21.95	23.59	22.22	22.29	22.63	22.19	21.92	19.69	18.70	21.33
EG	Benzene	2.41	0.12	7.77	4.99	8.52	7.78	5.05	8.12	7.80	5.14	5.86	8.39
	Toluene	30.77	25.51	33.25	32.54	28.75	29.84	32.61	27.91	31.00	28.85	25.12	28.35
	Xilene	27.83	23.13	26.97	29.10	24.59	24.20	27.66	23.38	25.45	27.56	24.61	27.42
